# Beliefs and practices regarding childhood fever among parents: a cross-sectional study from Palestine

**DOI:** 10.1186/1471-2431-13-66

**Published:** 2013-04-28

**Authors:** Sa’ed H Zyoud, Samah W Al-Jabi, Waleed M Sweileh, Masa M Nabulsi, Mais F Tubaila, Rahmat Awang, Ansam F Sawalha

**Affiliations:** 1 Poison Control and Drug Information Center (PCDIC), College of Medicine and Health Sciences, An-Najah National University, Nablus, Palestine; 2 Department of Pharmacology and Toxicology, College of Medicine and Health Sciences, An-Najah National University, Nablus, Palestine; 3 WHO Collaborating Centre for Drug Information, National Poison Centre, Universiti Sains Malaysia (USM), Penang, Malaysia; 4 Department of Clinical Pharmacy and Pharmacotherapy, College of Medicine and Health Sciences, An-Najah National University, Nablus, Palestine; 5 PharmD program, College of medicine and health sciences, An-Najah National University, Nablus, Palestine

**Keywords:** Children, Fever management, Belief, Temperature

## Abstract

**Background:**

Fever is an extremely common occurrence in paediatric patients and the most common cause for a child to be taken to the doctor. The literature indicates that parents have too many misconceptions and conflicting information about fever management. The aim of this study was to identify parents’ beliefs and practices regarding childhood fever management.

**Methods:**

We conducted a cross-sectional survey among parents whose children were enrolled and presented for health care at primary health care clinics in the Nablus region of Palestine. Data were collected using structured questionnaire interviews with parents. The questionnaire consisted of ‘yes/no’ responses and multiple-response questions. Descriptive statistics were used.

**Results:**

Overall, 402 parents were interviewed. All parents believed that fever could cause at least one harmful effect if left untreated. The harmful effects most frequently reported by parents were brain damage (38.1%), dehydration (15.7%), and other organs damage such as liver and kidney damage (14.2%). The study showed that 65.4% of parents would recognise fever by only touching the child, 31.6% would measure the temperature and 3.0% would assess temperature by touching and measuring the child. Antipyretic was preferred to be used by 34.8% of parents, while 49.8% stated that they preferred cold sponges, and 3.2% stated that they preferred homeopathic methods to treat fever. The most common factors influencing frequency of medication administration included physician’s instruction (61.7%), the degree of elevated temperature (14.9%) and instructions on the medication leaflet (13.7%). Of the participant parents, 53.2% believed antipyretics used to reduce fever were harmful. Parents reported the most harmful outcomes from these antipyretics to be allergic reactions (20.9%), effects on the stomach (16.9%), kidney damage (16.2%) and overdose (11.4%).

**Conclusions:**

Parents were anxious when dealing with a feverish child, which resulted in incorrect or inappropriate practices. Parents require reliable evidence-based information about the care of feverish children. These results indicate a need to develop and evaluate educational programs in our setting that will provide parents with education on fever and fever management.

## Background

Although fever was considered as a protective response for thousands of years, and was even induced by physicians to combat certain infections, the using of antipyretic drugs has led to the common belief that fever is maladaptive and harmful [1]. Studies have revealed that parents have several misconceptions and beliefs about fever, its role in illness, and its management [2,3]. Moreover, racial/ethnic variations exist in beliefs and practices regarding childhood fever [4]. Parents' fever phobia, as well as concern about and inappropriate treatment of childhood fever, are well documented and possibly caused by multiple factors [5]. Parents' ratings of the harmful effects of fever have changed from past to present, although their main concerns continue to be brain damage, febrile convulsions and death [2,5,6]. Principi et al. [7] stated that parents of febrile children take time off work, seek medical advice, purchase pharmaceuticals and need more assistance at home. Furthermore, Walsh et al. [8] documented that childhood fever has a socioeconomic, physical and emotional effect on parents.

To the best of our knowledge, although many reports related to care of a febrile child among different populations in the world have been published [1,4,6,8-18], none has been conducted in Palestine. Despite the fact that childhood fever management is receiving increasing attention regarding its prominent role in health care, little is known about Arab parents’ childhood fever management [1,19].

This study is designed to identify parents’ beliefs and practices regarding childhood fever management. We aim to provide a documented background about parents’ practices in the management of fever in their children, with the intention that this provides the basis for a large, comprehensive future study aimed at educating parents about the best methods of fever management and accurate dosing and using antipyretics. Furthermore, this study will be unique in that we probe extensively into parental understanding of the role of fever in illness, the use of antipyretics, and the knowledge and beliefs of parents about normal temperature and fever values.

## Methods

### Study design and area

A cross-sectional study design was used to address the research goals. The study was conducted in Nablus city with the surrounding camps and villages, with a population of 187839 in the city alone, and with a total population including Nablus city with the three Palestinian refugee camps and surrounding villages of 340117. Nablus district was chosen as it is considered one of the largest districts in Palestine and because of the convenience of the investigators who are working in this area. Around 47% of the population are children aged 0–17 years, while 28.1% are adults aged 18–35 years. The total number of families is 58750 and the average family size is 5.4 members. Furthermore, 15.9% of women and 67.4% of men are members of the workforce, with 12.9% of the population being unemployed [20].

### Study setting

Study participants were recruited from the most populated areas. We collected data from two destinations: health care centres for children, where parents went periodically to vaccinate their children and therefore where we focused on dates when we would be able to recruit the required sample, and from Rafedia Governmental Hospital, where we focused on paediatric outpatient clinics. The inclusion criteria for our study were: parents aged 19–48 years, who had at least one child aged between six months and six years, who were willing to participate, and who had given verbal consent to participate in the study [21]. The sample represents the general population of preschool children living in the study area from which information was collected.

### Sample size

The mean monthly number of parents with children who attend the primary clinic in Nablus city (study area population) is 39,400. This number was used as a guide to calculate the sample size needed for this study. By assuming a response distribution for temperature more than 38°C at which parents gave antipyretics was 50%, and allowing 5% margin of error at 95% confidence interval, the required sample size for the study was determined using Raosoft sample size calculator (http://www.raosoft.com/samplesize.html). The minimum effective sample size estimated for the survey was 377. In order to minimise erroneous results and increase the study reliability, the target sample size was increased to 402 participants. Therefore, a convenience sample of 402 respondents was identified between July 2012 and October 2012.

### Ethical approval

This study received approval from the Palestinian Ministry of Health and Institutional Review Board (IRB) at An-Najah National University. Verbal consent was also obtained from the parents prior to commencement of the study. The IRB considered waiving the requirement to obtain verbal consent for our protocols that were clearly below minimal risk and the research did not involve any therapeutic intervention.

### Data collection form

Subjects were interviewed by use of a questionnaire, developed on the basis of other previous similar concepts [1,4,6,8,11,12,22]. A questionnaire was developed to obtain socio-demographic information, such as the respondent’s age, gender, employment status, residency, number of children, income, years of education, and teaching and health care insurance coverage. For the parental survey, the questionnaire was developed to elicit information about definition of fever, concerns about fever and fever management. The questionnaire consisted of ‘yes/no’ responses and 15 multiple-response questions. Additional information included methods and frequency of temperature monitoring, methods used for body temperature control, and beliefs regarding potential consequences of fever. Furthermore, additional information included daily frequency of antipyretic administration, items addressing parents’ antipyretic use, influences on the use of antipyretics and perceptions of the safety of antipyretics used for fever management.

### Data collection procedure

Data collection was carried out by face-to-face interviews with the parents by principal investigators who were qualified clinical pharmacists. A total of 402 parents were eligible and were included in the final analysis. Regular evaluations took place throughout the abstraction period to identify any problems in data collection, interpretation of definitions, and application of study criteria. Before commencing data analysis, an extensive series of checks were performed for data consistency, proper sequences of data, and an evaluation of missing or incomplete data. The data collection was pre-tested in through a pilot study of 10 parents who were not included in the final analysis to check for the understandability and language clarity of questions, and all valid comments were taken into consideration by the principal researchers in the main survey, and the modified version was reviewed by experts to ensure content and construct validity.

### Statistical analysis

Data were entered and analysed using the Statistical Package for Social Sciences (SPSS; SPSS Inc., Chicago, IL, USA) program version 15. Descriptive statistics were used to describe the data; continuous data are presented as mean ± standard deviation (SD), and categorical data are expressed as numbers with percentages.

## Results

### Demographic data

A total of 402 completed questionnaires were evaluated. Table 1 shows the distribution of the socio-demographic characteristics of the parents who participated in the study. The mean age (± SD) of the parents was 37.4 ± 4.6 years and they had 1.4 ± 0.7 children aged under six years. Most of the participants were fathers (60.0%), had a village residency (86.0%), and were educated to university level or higher (56.2%). Most of the parents interviewed had a family income equal to or less than 1000 Jordanian Dinars.

**Table 1:** Socio-demographic data of parents participating in the study (N = 402)

**Variable**	**Item**	**Frequency (%) N = 402**
**Gender**	Male	241 (60.0)
	Female	161 (40.0)
**Number of children aged less than six years**	1 child	289 (71.9)
	2 children	79 (19.6)
	3 children	24 (6.0)
	4 children	10 (2.5)
**Health insurance**	Governmental insurance	163 (40.6)
	Private insurance	56 (13.9)
	Both	13 (3.2)
	Do not have one	170 (42.3)
**Father’s educational level**	Elementary school (primary)	23 (5.7)
	Middle school (junior high school)	42 (10.4)
	High school (secondary school)	112 (27.9)
	University	225 (56.0)
**Mother’s educational level**	Elementary school (primary)	17 (4.2)
	Middle school (junior high school)	44 (10.9)
	High school (secondary school)	133 (33.1)
	University	208 (51.8)
**Employment status**	Both works	108 (26.8)
	One of them works	278 (69.2)
	Neither works	16 (4.0)
**Income level of the family**^**a**^	Low (less than 500 JD)	89 (22.1)
	Average (500–1000 JD)	225 (56.0)
	High (1001–3000 JD)	78 (19.4)
	Very high (more than 3000 JD)	10 (2.5)
**Residency**	City	346 (86.0)
	Rural	40 (10.0)
	Palestinian refugee camps	16 (4.0)

^a^1 Jordanian Dinar (JD) equals 1.41 US Dollar.

### Parents' level of belief and understanding about the role of fever in illness

As shown in Table 2, the majority of parents (77.4%) believed that the symptoms of certain illnesses can cause fever in children, and 19.4% of parents believed that the cause of this was a natural result of child growth. Parents believed that the temperature representing fever ranged from 37 to 41°C. To enable comparisons between the literature and parents’ definitions of fever, temperatures 36.0–37.9°C were considered normal temperature and more than 38.0°C were considered fever [8,23]. Fever (temperature of 38.0–39.0°C) was reported by 78.3%; however, 7.0% identified a temperature of below 38.0°C as indicative of fever. Parents believed that fever could be harmful. The most frequent potentially harmful reasons for fever reported by parents were brain damage (38.1%), dehydration (15.7%), and other organs damage such as liver and kidney damage (14.2%).

**Table 2:** Beliefs about fever and harmful effects as reported by parents (N = 402)

**Variable**	**Frequency (%)**
**Beliefs about causes of fever**	
Fever is a symptom for certain illnesses	311 (77.4)
Fever is a natural result of child growth	78 (19.4)
Fever is a disease rather than a symptom	13 (3.2)
**Beliefs about temperature of feverish child**	
Less than 38°C	28 (7.0)
38–38.5°C	160 (39.7)
38.5-39°C	155 (38.6)
More than 39°C	59 (14.7)
**Beliefs about harmful effects of fever**	
Dehydration	63 (15.7)
Brain damage	153 (38.1)
Other organs damage (e.g. liver and kidney damage)	57 (14.2)
Indication of serious illness	19 (4.7)
Loss of consciousness	56 (13.9)
Febrile seizure	16 (4.0)
Brain damage + other organs damage + Indication of serious illness	26 (6.5)
All effects	12 (3.0)

### Parents’ methods of self management of a feverish child

As shown in Table 3, parents used different methods for child fever recognition. The study showed that 65.4% of parents recognised fever by only touching the child, approximately one third (31.6%) would measure the temperature, and 3.0% evaluated temperature by touching and measuring. The most common site that parents used for measuring temperature was the mouth (50.2%), followed by the anus (25.9%) and then the armpit (21.1%). In the present study, 34.8% of parents stated that they preferred to use antipyretic medicines, acetaminophen, or ibuprofen or diclofenac to treat fever if there were no co-morbid symptoms. Of particular note, 86.2% of parents stated that they would give their child antipyretic medicines for temperatures between 38–39°C. In addition to antipyretic medicines, 49.8% of parents stated that they proffered cold sponges, and 3.2% stated that they preferred homeopathic methods to treat fever (Table 3). When a child was feverish with co-morbid symptoms such as vomiting or diarrhoea, 50.2% of parents stated that they consulted a pharmacist, while 21.1% of the parents gave antipyretics and also consulted a physician. As shown in Table 3, the majority of parents (46.8%) stated that the source of antipyretics used were prescribed medications; the use of previous prescriptions for the same child and over-the-counter medicines were reported by 24.9% and 11.9% of parents, respectively.

**Table 3:** Parents’ methods for managing childhood fever (N = 402)

**Variable**	**Frequency (%)**
**Fever recognition**	
Touching child	263 (65.4)
Measuring temperature	127 (31.6)
Touching and measuring	12 (3.0)
**Site (method) used for measuring temperature**	
Mouth (oral)	202 (50.2)
Anus (rectal)	104 (25.9)
Armpit (axillary)	85 (21.1)
Other	11 (2.7)
**Temperature at which to give antipyretics**	
Less than 38°C	6 (1.5)
38–38.5°C	147 (36.6)
38.5-39°C	155 (38.6)
38–39°C	4 (1.0)
More than 39°C	90 (22.4)
**Fever management (if no co-morbid symptoms present)**	
Antipyretic use	140 (34.8)
Cold sponges	200 (49.8)
Homeopathic methods	13 (3.2)
Antipyretic + cold sponges	37 (9.2)
Other	12 (3.0)
**Fever management (if there is a co-morbid symptoms such as vomiting, diarrhea)**	
Antipyretic use and temperature monitoring	34 (8.5)
Antipyretic use and consult a physician	146 (36.3)
Seek physician assistance	9 (2.2)
Consult a pharmacist	209 (52.0)
Other	4 (1.0)
**Source of antipyretics used**	
Prescribed medications	188 (46.8)
Previous prescriptions for the same ill child	100 (24.9)
Previous prescriptions for one of the ill child’s siblings	18 (4.5)
Over-the-counter	48 (11.9)
Other	12 (3.0)

### Beliefs and practices influencing antipyretic use

As shown in Table 4, the decision to use antipyretics was influenced by temperature in 31.3% of parents. Furthermore, many parents (68.7%) cited additional factors which influenced their decision to use antipyretics. These factors included ineffectiveness of non-pharmacological or homeopathic methods (13.4%), presence of pain or discomfort (12.7%), and not eating or drinking (6.0%). Medications were also used when the child had a history of febrile convulsions (11.9%), presence of sleeping problems (4.7%) or presence of illness symptoms (10.4%). Furthermore, parents with multiple-responses to factors which influenced their decision to use antipyretics are shown in Table 4.

**Table 4:** Beliefs and practices influencing antipyretic use for managing childhood fever as reported by parents (N = 402)

**Variable**	**Frequency (%)**
**Decisions to use medications were primarily influenced by**	
To reduce temperature only when elevated	126 (31.3)
Presence of pain or discomfort	51 (12.7)
Presence of illness symptoms (e.g. vomiting, cough, cold)	42 (10.4)
Sleeping problems	19 (4.7)
Not eating or drinking	24 (6.0)
Presence of a history of febrile convulsions	48 (11.9)
Non-pharmacological or homeopathic methods were ineffective	54 (13.4)
Sleeping problems + not eating or drinking + non-pharmacological methods were ineffective	26 (6.5)
All factors	12 (3.0)
**Daily maximum frequency of antipyretic usage**	
1	30 (7.5)
2	126 (31.3)
3	118 (29.4)
4	109 (27.1)
5	5 (1.2)
6	14 (3.5)
**Factors influencing frequency of administration**	
Instructions on drug leaflet	55 (13.7)
Physician’s instructions	248 (61.7)
Pharmacist’s instructions	15 (3.7)
Severity of the accompanying disease	12 (3.0)
Degree of elevated temperature	60 (14.9)
Child’s weight	4 (1.0)
Child’s age	8 (2.0)
**Factors influencing antipyretic dose**	
Drug instructions on leaflet	60 (14.9)
Physician’s instructions	221 (55.0)
Pharmacist’s instructions	31 (7.7)
Severity of accompanying disease symptoms	8 (2.0)
Child’s age	31 (7.7)
Child’s weight	8 (2.0)
Degree of temperature elevation	12 (3.0)
Child’s inactivity	1 (0.2)
Drug instructions on leaflet + Child’s weight	30 (7.5)
**Preferred antipyretics pharmaceutical dosage form**	
Syrups	206 (51.2)
Suppositories	63 (15.7)
Injections	6 (1.5)
Syrups and suppositories combined	127 (31.6)
**Difficulties experienced during administration of medications**	
Children refusing to swallow the medication	197 (49.0)
Children spitting it out	128 (31.8)
Children being too distressed by the illness/fever	46 (11.4)
Children being too sleepy	18 (4.5)
Children refusing to swallow the medication + children spitting it out	13 (3.2)
**Procedure to ensure that febrile children received their medications**	
Used force	19 (4.7)
Coaxed and encouraged their child	269 (66.9)
Mixed the medication with foods or drinks	44 (10.9)
Sought medical advice	10 (2.5)
Gave suppositories instead of syrup	50 (12.4)
Used non-pharmacological methods	10 (2.5)
**Beliefs about the harmful outcomes associated with antipyretic use**	
Liver damage	60 (14.9)
Overdose	46 (11.4)
Kidney damage	65 (16.2)
Effect on stomach	68 (16.9)
Immunity suppression	7 (1.7)
Allergic reactions	84 (20.9)
Other	72 (17.9)

When a child was feverish, oral medications were preferred by 51.2% of parents, whereas 15.7% preferred rectal medications and 31.6% preferred both. In response to a question concerning frequency of medication administration, the majority of parents reported administering antipyretics twice daily (31.5%). Although some parents (27.1%) reported administration of medication at four hourly intervals, 4.7% reported more frequent intervals. The most common factors influencing frequency of medicine administration included physicians' instructions (61.7%), degree of elevated temperature (14.9%), and instructions on the medication leaflet (13.7%). Additionally, the most common factors influencing the dose of antipyretics were physicians' instructions (55.0%), and instructions on the medication leaflet (14.9%); (Table 4).

Feverish children are not always compliant with administration of antipyretics; almost two-thirds of parents (65.9%) had experienced difficulties giving their children medication. These included children either refusing to swallow the medication or spitting it out (49.0% and 31.8%, respectively). To ensure feverish children received medications, parents used a different administration method such as encouraging their child (66.9%) or giving suppositories instead of syrup (12.4%) or mixing the medication with foods or drinks (10.9%). Of all participant parents, 53.2% believed antipyretic use to reduce fever was harmful. Parents reported the most harmful outcomes from these antipyretics to be allergic reactions (20.9%), effect on the stomach (16.9%), kidney damage (16.2%), and overdose (11.4%); (Table 4).

## Discussion

The present study is an analysis of current beliefs about fever and practices in childhood fever management in a large sample of parents. The concept of our study is novel to the Palestinian population. It is a one part of social sciences that is often neglected by researchers in the area. Although health, literacy and education currently have a higher standard in the Israeli-occupied Palestinian territory than in several Arab countries, 52% of families have been reported to live below the national poverty line of US$ 3.15 per person per day [24]. In contrast with the decline between year 1967 and 1987, infant mortality stalled at around 27 per 1000 between year 2000 and 2006, the same as that reported in the 1990s, which suggests a slowdown of health improvements, a possible increase in health disparities, or an indication of deteriorating conditions [24]. Around two thirds of parents recognise fever in their child by non-measurement methods such as observing by touching the child. This practice of fever determination has been shown to be inaccurate with a high percentage of false-negatives or false-positives [25]. Furthermore, Chaturvedi [26] found that the touching method is not a valid screening test for fever. Jalil et al. [1] stated that measuring the temperature is obviously the most accurate method of detecting fever but these authors found that only one third of mothers actually measured the child’s temperature at home to detect fever.

Body temperature is one of the most widespread clinical signs used by mothers to make a decision about whether a child is ill [21]. The results of our survey show that parents generally prefer the oral method for taking their child’s temperature. The accuracy of oral temperature measurement is influenced by the ability of the patient to cooperate, recently ingested hot or cold liquid or food, tachypnoea, and location and length of time of the thermometer in the mouth [27]. The rectal method is considered more reliable and sensitive than the oral route; however, some consider it inappropriate for parents to use because of the risk of the thermometer breaking, rectal injury, and cross-infections [21]. Parents define temperatures between 38.0 and 39.0°C as fever. This result is consistent with the most commonly reported level used for fever determination [1,5,28,29]. Fever is defined as a temperature above the normal range. A rectal temperature of 38.0°C or more, an axillary temperature of 37.2°C or more, and an oral temperature of 37.5°C or more are all considered to be indicative of fever [19,30]. Furthermore, temperatures 36.0–37.9°C were considered normal, 38.0–39.0°C mild fever, 39.1–40.4°C high fever and 40.5°C and above very high fever [8,23].

The most frequent potentially harmful reasons for fever reported by parents are brain damage, other organs damage, dehydration and convulsion. These parents, similar to their international counterparts, believe fever to be harmful and to cause brain damage, febrile convulsions and dehydration [1,8,31]. Walsh et al. [8] concluded that education about the prevalence and prognosis of febrile convulsions and safe caring of a child during a febrile convulsion is needed and may contribute to reducing fever phobia and unnecessary fever reduction.

The most commonly reported methods to treat feverish child are sponging and giving antipyretic medication. Jalil et al. [1] stated that research literature confirmed that sponge bathing is ineffective and causes shivering, which increases the body’s temperature, as the hypothalamus attempts to offset the decrease in body temperature produced by sponging [32]. This method is effective in the short term, but increases the child’s discomfort and encourages temperature-conserving behaviour and as a result, cannot be recommended for feverish children. Additionally, current paediatric practice for a febrile child includes the use of antipyretics for a temperature greater than 38.5°C. The main indication for prescribing antipyretics is not to decrease the temperature, but to relieve the parents’ anxiety and thereby the child’s discomfort [1]. Impicciatore et al. [21] stated that parents might not know that many physicians had agreed to fever reduction measures because the child might be uncomfortable or because the parents were anxious, and not because of potential complications.

These beliefs indicate an urgent need for health care professionals to educate parents about the effects of antipyretics. The belief of parents regarding the magical qualities of antipyretics encourages their use, increases the probability of overdosing, and increases fever phobia. There is a need to provide parents with appropriate fever management strategies, such as giving their febrile children more fluids and rest, and keeping them comfortable, as well as guidelines of when to use medications and seek medical advice, in order to reduce fever phobias and the probability of overdosing [22]. Furthermore, Walsh et al. [22] stated that increased use of antipyretics could lead to an associated increase in accidental overdosing. Over-the-counter medications are readily available, and marketing includes recommendations from health care professionals, especially physician and pharmacists, who have a responsibility in child advocacy to make parents more skilled in the use of such easily available and potentially harmful drugs [22].

There were some limitations to this study that deserve further discussion. Our results may not be generalized to all Palestinian population. Furthermore, data regarding daily maximum doses of antipyretics were missing from the study. Another limitation was the cross-sectional design of the study, which examined the associations between variables at one point in time, rather than longitudinally. In addition, because of the cross-sectional design of the study, we relied on parental information, and findings may be limited by recall bias.

## Conclusion

This study has shown that parents are often unaware of the level of body temperature that indicates a fever and the way that they deal with a feverish child is sometimes incorrect or inappropriate. Parents believe that fever may lead to serious complications and this probably leads to an increased probability of overdosing and an increase in fever phobia. Furthermore, Palestinian parents have similar concerns about harmful outcomes from childhood fever as their international counterparts. Parents require reliable evidence-based information about the care of feverish children. These results indicate a need to develop and evaluate educational programs in our setting that will provide parents with education on fever and fever management. Thus, replication of this study in other Palestinian states or Arab countries is recommended to determine the extent of parental fever phobia and medication misuse in different cultures.

## Abbreviations

IRB: Institutional Review Board; SPSS: Statistical Package for Social Sciences; SD: Standard deviation.

## Competing interests

The authors declare that they have no competing interests.

## Authors’ contributions

All authors were involved in drafting the article, and all authors approved the final version to be submitted for publication. SZ conceived of the study conception and design, organized and supervised the data collection, and provided analysis, interpretation, and writing. SA and WS participated in the study design, and provided critical revision of manuscript for important intellectual content. MN and MT carried out the data collection and results tabulation. AS participated in the study design. RA was involved in concept and editing the manuscript.

## Pre-publication history

The pre-publication history for this paper can be accessed here:

http://www.biomedcentral.com/1471-2431/13/66/prepub
